# The biochemical properties of the two *Arabidopsis thaliana* isochorismate synthases

**DOI:** 10.1042/BCJ20161069

**Published:** 2017-04-28

**Authors:** Keith M. Macaulay, Geraldine A. Heath, Alessio Ciulli, Alex M. Murphy, Chris Abell, John P. Carr, Alison G. Smith

**Affiliations:** 1 Department of Plant Sciences, University of Cambridge, Downing Street, Cambridge CB2 3EA, U.K.; 2 University Chemical Laboratory, University of Cambridge, Lensfield Road, Cambridge CB2 1EW, U.K.

**Keywords:** chloroplasts, plant hormones, salicylic acid

## Abstract

The important plant hormone salicylic acid (SA; 2-hydroxybenzoic acid) regulates several key plant responses including, most notably, defence against pathogens. A key enzyme for SA biosynthesis is isochorismate synthase (ICS), which converts chorismate into isochorismate, and for which there are two genes in *Arabidopsis thaliana*. One (*AtICS1*) has been shown to be required for increased SA biosynthesis in response to pathogens and its expression can be stimulated throughout the leaf by virus infection and exogenous SA. The other (*AtICS2*) appears to be expressed constitutively, predominantly in the plant vasculature. Here, we characterise the enzymatic activity of both isozymes expressed as hexahistidine fusion proteins in *Escherichia coli.* We show for the first time that recombinant AtICS2 is enzymatically active. Both isozymes are Mg^2+^-dependent with similar temperature optima (ca. 33°C) and similar *K*_m_ values for chorismate of 34.3 ± 3.7 and 28.8 ± 6.9 µM for ICS1 and ICS2, respectively, but reaction rates were greater for ICS1 than for ICS2, with respective values for *V*_max_ of 63.5 ± 2.4 and 28.3 ± 2.0 nM s^−1^ and for *k*_cat_ of 38.1 ± 1.5 and 17.0 ± 1.2 min^−1^. However, neither enzyme displayed isochorismate pyruvate lyase (IPL) activity, which would enable these proteins to act as bifunctional SA synthases, i.e. to convert chorismate into SA. These results show that although *Arabidopsis* has two functional ICS enzymes, it must possess one or more IPL enzymes to complete biosynthesis of SA starting from chorismate.

## Introduction

Salicylic acid (SA; 2-hydroxybenzoic acid) is an important plant hormone [1]. Although it has been most thoroughly studied with respect to its functions in pathogen resistance, SA sits within a complex regulatory network affecting signalling by other phytohormones and it directly or indirectly affects a wide range of plant responses [1]. SA biosynthesis is triggered by multiple stimuli, including abiotic stress [2], pathogen infection [3–5] and developmental cues [6–8]. The process by which plants synthesise SA is still not fully understood. Early studies in tobacco pointed to a role for the phenylpropanoid pathway, with benzoic acid as the immediate metabolic precursor to SA [9]. Subsequently, the discovery of the *SA induction-deficient 2* (*sid2*) mutant in *Arabidopsis thaliana* (hereafter referred to as *Arabidopsis*) and the identification of isochorismate synthase 1 (ICS1; AtICS1:EC 5.4.4.2, also known as isochorismate hydroxymutase) as the enzyme encoded by the wild-type *SID2* allele showed that, in this plant, SA is synthesised from chorismic acid, derived from the shikimic acid pathway, and that SA biosynthesis occurs in the plastid [5,10–12]. In some plants, as shown in soybean (*Glycine max*), there can be redundancy in SA biosynthesis with both the shikimic acid pathway and the phenylpropanoid pathway being required to generate sufficient SA for effective pathogen defence [13]. However, in *Arabidopsis*, SA biosynthesis appears to be solely dependent on the shikimic acid pathway and follows a similar route to that which occurs in bacteria.

In bacteria, SA is a common metabolite that is an intermediate in the biosynthesis of siderophores: compounds that are used by bacteria to scavenge iron. There are two known bacterial routes for SA biosynthesis that utilise chorismate. In some bacteria, exemplified by *Yersinia enterocolitica*, the *Irp9* product is a bifunctional SA synthase (SAS), which can convert chorismate into isochorismate (ICS activity) and isochorismate into SA (isochorismate pyruvate lyase, IPL) [14,15]. In contrast, other bacteria possess separate ICS and IPL enzymes. For example, the *Pseudomonas aeruginosa* genes *PchA* and *PchB* encode proteins with, respectively, ICS and IPL (EC 4.2.99.21) activities [16]. *Escherichia coli* does not make SA, but it encodes two ICS enzymes EntC and MenF that are involved in siderophore biosynthesis (see ref. [17] and references therein). Engineering of transgenic *Arabidopsis* to express either a bifunctional SAS [18], or separate ICS and IPL enzymes [19], yielded plants that constitutively overproduced SA. These experiments did not reveal whether or not plants naturally possess a bifunctional SAS or distinct ICS and IPL enzymes, but they indicated that, in principle, either arrangement might be functional in plants.

SA biosynthesis is elevated dramatically in plants following attack by avirulent pathogens that induce a hypersensitive response (HR). The HR is a form of resistance in which pathogen spread beyond the initial site of penetration is prevented. During the HR, SA accumulates initially in the vicinity of the pathogen entry site, but subsequently its levels increase throughout the plant [3,4,10,20,21]. However, in some cases, increased SA accumulation can accompany the spread of virulent pathogens that are capable of overcoming or evading host resistance (e.g., see ref. [22]). Increased SA accumulation radically remodels plant gene expression and induces systemic acquired resistance, an enhanced state of resistance against a very wide range of pathogens [23].

*Arabidopsis* encodes two *ICS* genes, as does soybean, whereas other higher plants with sequenced genomes, such as *Populus* (poplar), *Oryza sativa* (rice), *Ricinus communis* (castor bean), *Vitis vinifera* (grapevine) and *Medicago truncatula* (alfalfa), possess a single gene [24]. The isozyme encoded by *AtICS1* (originally identified as *SID2*) is responsible for the major proportion of SA biosynthesis in plants during responses to pathogens [5]. Increased accumulation of the *AtICS1* transcript can be caused by pathogen attack or by treatment with exogenous SA [5,10,12,25], but previous analysis suggested that *AtICS2* behaves differently in that its expression is not responsive to these stimuli [25]. In this work, we investigated expression of the two *Arabidopsis ICS* genes in more detail within the plant. In addition, we purified recombinant forms of the enzymes to investigate whether they had different catalytic properties and establish whether one or either of them had IPL activity.

## Experimental

### Bioinformatics

Amino acid sequences were aligned by CLUSTALW [26] and ESPript [27], trimmed to remove any predicted transit peptides [28] and inspected manually. iTASSER [29] was used for homology modelling of ICS1 and ICS2 using all known protein structures in the Protein Data Bank (PDB; http://www.pdb.org).

### Molecular cloning of *A. thaliana AtICS1* and *AtICS2*

To clone the two ICS protein-coding sequences from *Arabidopsis*, PCR was used to amplify sequences from cDNA templates. For *AtICS1*, a 4-week-old *Arabidopsis* seedling cDNA library in the yeast expression vector pFL61 [30] was used. For amplification of *AtICS2*, the use of the pFL61 library proved unsuccessful, so cDNA synthesised from the *genomes uncoupled 4* (*gun4*) mutant of *Arabidopsis* [31] was used as a template instead, as this displayed elevated levels of expression of *AtICS2* over wild type in microarray datasets [32].

Oligonucleotide primers (ICS1 F: 5′-ATCGTCGACCCATATGAATGGTTGTGATGGA-3′; ICS1 R: 5′-ATCGTCGACTCAATTAATCGCCTGTAGAGA-3′; ICS2 F: 5′-ATCGTCGACCCATATGAACGGATGTGAGGCT-3′; ICS2 R 5′-ATACTCGAGTTAGTTGATTGGTTGC-3′) were designed to introduce unique restriction sites (shown underlined: 5′ *NdeI* and 3′ *SalI* for ICS1; 5′ *NdeI* and 3′ *XhoI* for ICS2) for later subcloning, and to amplify the two ICS coding sequences without the presence of the transit peptide sequences. Since ICS2 contained an endogenous thrombin cleavage site, a modified version of pET-28a (+), pET-28a TEV (+) was used that has a tobacco etch virus (TEV) protease recognition sequence in place of the thrombin sequence. PCR products were digested with the appropriate restriction enzymes (*NdeI* and *SalI* for ICS1; *NdeI* and *XhoI* for ICS2) and were ligated into pET-28a TEV. Clones were identified and the coding sequence was verified to be in-frame with the His-tag by sequencing; plasmids were called pHis-ICS1 and pHis-ICS2, for His_6_-AtICS1 and His_6_-AtICS2 constructs, respectively. Routine molecular biology techniques and bacterial growth media used in this work are described by Maniatis et al. [33]*.* The generation of transgenic *Arabidopsis* plants harbouring *AtICS* promoter*-*β*-glucuronidase* (GUS) reporter gene constructs for *AtICS1* and *AtICS2*, and detection methods for GUS activity were described previously [22,25].

### Heterologous expression and purification of *AtICS1* and *AtICS2*

The plasmids pHis-ICS1 and pHis-ICS2 were used to transform the *E. coli* strain Rosetta™ DE3 pLysS (Merck, http://www.merck-chemicals.co.uk). Transformed cells were grown in 2xYT medium supplemented with 50 µg ml^−1^ kanamycin, 34 µg ml^−1^ chloramphenicol and 0.2% w/v glucose. A 50 ml overnight starter culture grown at 37°C was used to inoculate 1 l of fresh medium, and ICS protein synthesis was induced at an OD_600_ of 0.6 by introducing isopropyl β-d-thiogalactopyranoside (IPTG) to a final concentration of 1 mM, and ethanol was also added to a final concentration of 3% w/v. The IPTG-induced culture was grown for 24 h at 16°C before cells were collected by centrifugation and stored at −80°C.

All further purification steps were performed at 4°C. Cell pellets were thawed and resuspended in resuspension buffer [20 mM potassium phosphate buffer (pH 7.4), 500 mM NaCl, 10% v/v glycerol, 1 mM DTT and 1% v/v Triton X-100] supplemented with Complete EDTA-Free Protease Inhibitor Cocktail (Roche, http://www.roche-applied-science.com). Cells were lysed by three passes through an Emulsiflex C-5 high-pressure homogeniser (Avestin, http://www.avestin.com) operated at 15 000 p.s.i. The lysate was clarified by centrifugation and filtered through a 0.45 µm pore diameter syringe filter (Sartorius, http://www.sartorius.com) prior to chromatography. Samples were applied to a 5 ml HisTrap HP chelating column (GE Healthcare, http://www.gehealthcare.com) at a flow rate of 0.75 ml min^−1^, which had previously been charged with 0.1 M NiSO_4_ and equilibrated with 10 column volumes of column buffer [20 mM potassium phosphate buffer (pH 7.4), 500 mM NaCl and 10% v/v glycerol] using an AktaFPLC system (GE Healthcare). Elution was performed using a linear gradient of column buffer containing 0.5 M imidazole over 10 column volumes at a flow rate of 1.0 ml min^−1^. Fractions containing protein were examined by SDS–PAGE and those fractions containing the correct-sized protein were combined and concentrated to <1 ml using a centrifugal concentrator (Sartorius). This concentrated sample was applied to a HiLoad 16/60 Superdex 200 preparative grade column (GE Healthcare), previously equilibrated with gel filtration buffer [50 mM Tris–HCl (pH 8.0), 500 µM EDTA, 10% v/v glycerol and 1 mM DTT], at a flow rate of 0.5 ml min^−1^. Fractions containing protein were examined by SDS–PAGE to determine purity, and those containing pure protein of the predicted molecular mass were pooled and concentrated using a centrifugal concentrator (Sartorius). Aliquots of His_6_-AtICS1 and His_6_-AtICS2 were flash-frozen in liquid nitrogen and stored at −80°C. The identity of the purified proteins was further verified by western blotting, electrospray mass spectrometry and activity assays.

### Heterologous expression and purification of His_6_-PchB

The *P. aeruginosa* IPL (PchB) was expressed in *E. coli* as an N-terminal hexahistidine fusion protein. The pHis-PchB plasmid was a gift from Dr Sridharan Sudharsan (University of Cambridge) and was used to transform *E. coli* BL21 (DE3) cells (Merck). These were inoculated into 100 ml 2xYT medium containing kanamycin and incubated overnight at 37°C. A 50 ml aliquot of this starter culture was then used to inoculate 500 ml of 2xYT medium containing 50 µg ml^−1^ kanamycin, and grown at 37°C until the OD_600_ reached 0.6. Expression of His_6_-PchB was induced by the addition of 1 mM IPTG for a period of 4 h. Cells were collected by centrifugation and stored at −80°C until needed. Protein purification was performed in a manner identical for His_6_-ICS1 and His_6_-ICS2, except for the compositions of the column buffer [20 mM potassium phosphate buffer (pH 7.4), 500 mM NaCl and 20 mM imidazole] and gel filtration buffer [50 mM potassium phosphate buffer (pH 7.0) and 150 mM NaCl]. Aliquots of purified protein were flash-frozen in liquid nitrogen and stored at −80°C. EDTA was removed from samples of His_6_-ICS1 and His_6_-ICS2 by passage through a Zeba Spin desalting column (Thermo Scientific, http://www.thermo.com) equilibrated with assay buffer [50 mM Tris–HCl (pH 8.0), 10% v/v glycerol and 1 mM DTT]. Chorismate for use in enzyme assays was a gift from Dr Nigel Howard (University of Cambridge) and had been prepared according to Grisostomi et al. [34].

### High-performance liquid chromatography detection of ICS activity

To detect the conversion of chorismate into isochorismate, reverse-phase HPLC (high-performance liquid chromatography) was used. A 400 µl mixture containing 100 mM Tris–HCl (pH 7.7), 10 mM MgCl_2_, 1.5 mM chorismate and 2.5 µM His_6_-ICS1 or His_6_-ICS2 was incubated at 30°C for 30 min. The reaction was stopped by centrifuging the mixture in an Amicon Ultra 0.5 ml 3 kDa centrifugal concentrator (Millipore, http://www.millipore.com). A 100 µl aliquot of the flow-through was injected onto the HPLC column. HPLC was performed using an Accela fast HPLC system with detection by a photodiode array (PDA) detector and a Surveyor FLPlus fluorimeter (Thermo Scientific). A Dynamax Microsorb 60-8 250 × 4.6 mm C18 column (Varian, http://www.varianinc.com) was used for separation at a flow rate of 1.0 ml min^−1^. Solvent A consisted of aqueous 0.1% v/v trifluoroacetic acid (TFA) and solvent B was acetonitrile +0.1% TFA. The gradient profile used was as follows: between 0 and 15 min, a linear increase from 5 to 18% B; from 15 to 18 min, a linear increase to 65% B and then isocratic for 5 min; a linear increase to 95% B over 7 min and then isocratic for 5 min; a linear decrease to 5% B over 5 min. The PDA detector measured absorbance at 274 nm, while the fluorimeter used an excitation wavelength of 305 nm and an emission wavelength of 407 nm. Under these conditions, isochorismate was eluted from the column after a retention time of 14.0 min, and then chorismate at 15.0 min, with salicylate at 21.1 min.

### ^1^H nuclear magnetic resonance assay for ICS activity

Independent analysis of the activity of AtICS1 and AtICS2 was carried out using ^1^H NMR (nuclear magnetic resonance) as described previously [15]. Data were collected on a Bruker Avance 700 MHz Ultrashield spectrometer equipped with a 5 mm triple TXI cryoprobe with *Z* gradients (Bruker UK Ltd, Coventry). Reaction mixtures consisted of 20 mM potassium phosphate buffer (pH 7.0), 5 mM MgCl_2_, 1.3 mM chorismate and 2.5 µM His_6_-ICS1 or His_6_-ICS2 and 10% v/v D_2_O. (Trimethylsilyl)-propionic acid-*d*4 (20 µM) was present for calibration purposes. ^1^H NMR spectra were obtained at 4 min, 15 min, 2 h and 40 h after starting the reaction and were recorded for 90 s at 293 K. The resulting spectra were analysed with TopSpin 2.0. δH (700 MHz, 90% H_2_O, 10% D_2_O): chorismate, 6.58 (1H, s, H2), 6.33 (1H, d, *J* 10.0 Hz, H6), 5.98 (1H, dd, *J* 10.0 and 2.5 Hz, H5), 5.22 (1H, d, *J* 3.0 Hz, H8b); isochorismate, 6.84 (1H, d, *J* 5.5 Hz, H6), 6.36 (1H, dd, *J* 9.5 Hz and 5.5 Hz, H5), 6.22 (1H, dd, *J* 9.5 and 4.5 Hz, H4) and 5.25 (1H, d, *J* 2.5 Hz, H8b).

### Coupled fluorimetric assay for ICS activity

To facilitate the collection of quantitative data for kinetic analysis, ICS activity was detected using a coupled assay essentially as previously described by Sridharan et al. [17]. The standard reaction mixture consisted of 100 mM Tris–HCl (pH 7.7), 10 mM MgCl_2_, 1 µM His_6_-PchB, 100 nM His_6_-ICS1 or His_6_-ICS2 and 75 µM chorismate. The reaction mixture and chorismate were aliquoted into the wells of a black FLUOTRAC™ 200 96-well flat-bottomed plate (Greiner, http://www.greinerbioone.com) and preincubated for 10 min at 30°C in a FLUOstar Optima plate reader (BMG Labtech, http://www.bmglabtech.com). Reactions were initiated in parallel using a multichannel pipette to add the chorismate substrate to each well simultaneously and mixed by pipetting. Fluorescence was quantified at an excitation wavelength of 300 nm and an emission wavelength of 420 nm. Reactions were performed in triplicate, and the mean and standard error of the mean were reported. For determination of the kinetic parameters *K*_m_, *V*_max_ and *k*_cat_, the standard reaction mixture was used with the following concentrations of chorismic acid: 0, 4, 8, 15, 25, 50, 100, 200 and 300 µM. Initial rates were calculated and plotted as a function of chorismate concentration. Curve fitting was performed by gnuplot v4 (http://www.gnuplot.info) and kinetic parameters were determined.

### Temperature and pH dependence of ICS activity

To obtain temperature optima for His_6_-ICS1 and His_6_-ICS2, the standard reaction mixture was used in a 300 µL reaction volume. Reaction mixtures were preincubated in 1.5 ml microcentrifuge tubes in a water bath for 10 min and transferred to a preincubated Starna micro-cuvette (OptiGlass Ltd; http://www.optiglass.com) containing chorismate. To measure fluorescence, an LS-55 fluorescence spectrometer (PerkinElmer; http://www.perkinelmer.com) was used, equipped with a Peltier block (PerkinElmer). Heating and cooling of the Peltier block was performed by a recirculating water bath, which was placed in a refrigerator for temperatures below ambient conditions. Activity was investigated at 12, 20, 25, 30, 37 and 42°C. To obtain pH optima for His_6_-ICS1 and His_6_-ICS2 activities, buffering for the reaction mixtures was provided by: 4-morpholineethanesulfonic acid for pH 5.0, 5.5, 6.0 or 6.5; 4-morpholinepropanesulfonic acid for pH 7.0 and 7.5; Tris–HCl for pH 7.5, 7.7, 8.0 or 8.5; 2-(cyclohexylamino)ethanesulfonic acid for pH 9.0, 9.5 or 10.0 and 3-(cyclohexylamino)-1-propanesulfonic acid for pH 11.0 [10].

## Results

### Arabidopsis *ICS1* and *ICS2* are probably the result of genome duplication

As reported by Yuan et al. [24], the structure of the two *Arabidopsis ICS* genes is very similar, with identical intron–exon positions, except that exon IV of *AtICS1* is substituted by two exons in *AtICS2*. The two genes are both located on chromosome 1 on different sides of the centromere (Supplementary Figure S1A), and inspection of the genomic context of the two genes revealed that they are likely to be the result of a duplication event, since they are bordered by similar genes. At the DNA sequence level, the protein-coding regions of the two genes share a high degree of similarity, but this does not extend into the untranslated regions. In addition, analysis of 5-methylcytosine sequencing datasets [35] revealed that there are potential methylation sites in the promoter of *AtICS2* but not in the *AtICS1* promoter (Supplementary Figure S1B). These features may explain the different *in planta* expression patterns of the two genes. Our previous work showed that application of exogenous SA to plants expressing the GUS reporter gene under the control of either the *AtICS1* or *AtICS2* promoters stimulated *AtICS1* expression, but not *AtICS2* [25]. GUS activity was also enhanced in *AtICS1*::GUS plants by infection with cucumber mosaic virus but not with tobacco mosaic virus [22]; however, the effects of infection on *AtICS2* expression were not studied. We therefore examined *AtICS2*::GUS plants (Supplementary Figure S2). The results confirmed that *AtICS2* promoter activity is not stimulated by virus infection. Moreover, closer inspection of the distribution of GUS activity within the plants revealed that *AtICS2* expression in leaf tissue appears to be localised most strongly in the vasculature and hydathodes.

### Structural features of *Arabidopsis* ICS1 and ICS2

**Figure 1.**
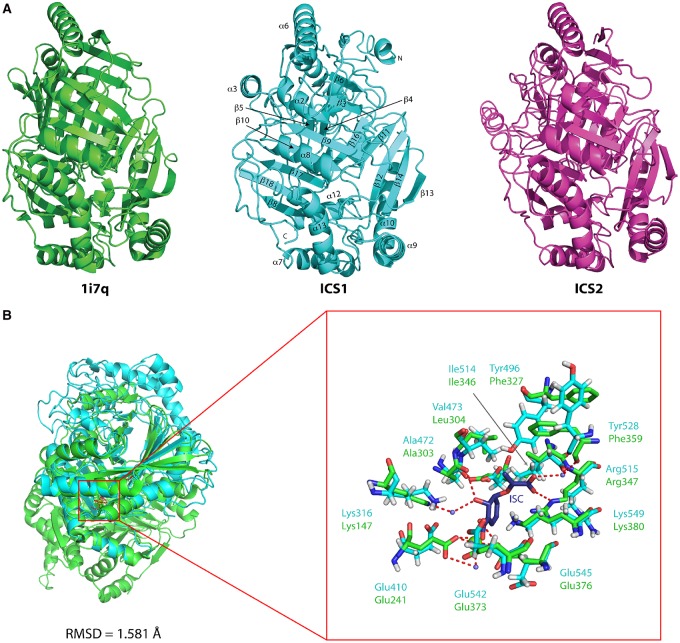
Homology models of *Arabidopsis* ICS1 and ICS2. (**A**) Primary amino acid sequences for the two *Arabidopsis* ICS isozymes were submitted to the iTASSER homology modelling server [29] for structural alignment with existing proteins structures. The reference structure, *S. marcescens* anthranilate synthase (TrpE; PDB accession 1I7Q), is shown alongside the models obtained with this structure for ICS1 and ICS2. α-helices and β-sheets are numbered according to the scheme shown in Supplementary Figure S3; some labels are omitted here for clarity. Amino (N) and carboxy (C) termini are labelled accordingly. ICS1 and ICS2 adopt the same overall structure, with identical arrangement of α-helices and β-sheets, as might be expected for two paralogous proteins. Images obtained using MacPyMOL (http://www.pymol.org). (**B**) Comparison of ICS1 (pale blue) with *E. coli EntC* (green), showing very similar active sites.

There has been extensive characterisation of bacterial isochorismate synthases and salicylate synthases, and X-ray crystal structures are known for EntC and other chorismate-utilising enzymes [17], revealing that they share many structural features. Alignment of the amino acid sequences of the mature *Arabidopsis* ICS proteins (i.e. without the chloroplast transit peptides) with those of bifunctional SAS enzymes from *Y. enterocolitica* and *Mycobacterium tuberculosis*, and the two ICSs from *E. coli* and another chorismate-utilising enzyme, anthranilate synthase TrpE (Supplementary Figure S3), reveal considerable conservation, particularly at the C-terminus. However, it is not possible to infer whether the *Arabidopsis* enzymes are likely to have IPL activity from this comparison. Accordingly, we carried out homology modelling of the *Arabidopsis* ICS proteins with these known structures using the iTASSER server [29]. Figure 1A shows the highest scoring models for AtICS1 and AtICS2 together with TrpE from *Serratia marcescens* (PDB accession 1i7Q). This was the structure automatically selected from the PDB database by iTASSER, rather than *bona fide* ICSs such as *E. coli* ICS proteins MenF and EntC, because of better overall structural (rather than sequence) similarity, particularly at the N-terminus. The TrpE structure provided 87% coverage for predicted structural elements for AtICS1 and AtICS2, versus 75 and 76% sequence similarity with MenF and EntC, respectively. The modelled structure of both *Arabidopsis* ICS proteins consists of an α/β-fold with an α-helical outer layer surrounding a core of β-sheets, as is often the case for chorismate-utilising enzymes [17]. It is very similar to that of *E. coli* EntC (PDB i.d. 3hwo) although there are two additional α-helices, α3, α4, which are absent from EntC.

By superimposing the AtICS1 model onto the EntC structure containing isochorismate bound within the active site [17], it is possible to infer where the AtICS1 active site might lie (Figure 1B; data shown only for AtICS1 since ICS2 was identical). The AtICS1 structure is shown in pale blue, and EntC in green, with the region of the active site enlarged in the inset. In terms of residues important in catalysis, EntC and AtICS1 are similar although with some conservative substitutions. Instead of a pair of phenylalanine residues (Phe327 and Phe359) stacking (as in EntC), two tyrosine residues (Tyr496 and Tyr528) may perform the same function in AtICS1, tethering β14 to β18 (β18 and β21 in EntC). Leu304 in EntC is substituted in AtICS1 by another branched-chain amino acid, Val473, which may have additional steric effects. Also, like EntC, and unlike any other chorismate-utilising enzymes, there is an additional α-helix (α2a in EntC, which is extended to form α6 in ICS1 and ICS2), shown at the top of the structures in Figure 1A.

### Expression of *AtICS1* and *AtICS2* in *E. coli*

The structural analysis suggests that both *Arabidopsis* ICS enzymes would have similar activity. To test this, hexahistidine-tagged recombinant AtICS1 and AtICS2 proteins were expressed in *E. coli* and purified. Several preliminary attempts to express the His_6_-AtICS2 protein failed, which might suggest that the conditions in the *E. coli* cytosol are unsuitable for high-level expression of this protein. However, it proved possible to express the proteins in *E. coli*, provided that the induction medium was supplemented with 3% (v/v) ethanol. The addition of ethanol is thought to increase expression of *E. coli* chaperones, which may aid correct folding and inhibit precipitation of overexpressed proteins [36]. Soluble His_6_-AtICS1 and His_6_-AtICS2 protein samples of high purity were obtained from *E. coli* lysates using a combination of Ni^2+^-affinity and size-exclusion chromatography to result in proteins of the expected size of ∼60 kDa (Supplementary Figure S4). The use of standards on the Superdex 200 column confirmed that both were monomeric. Western blotting of the purified fractions using antibodies against the hexahistidine tag confirmed that the ∼60 kDa bands corresponded to the recombinant proteins, whereas a minor contaminant of ∼29 kDa did not cross-react with the antibodies (labelled as II in Supplementary Figure S4B).

### ICS1 and ICS2 are both monofunctional isochorismate synthases but not salicylate synthases

**Figure 2.**
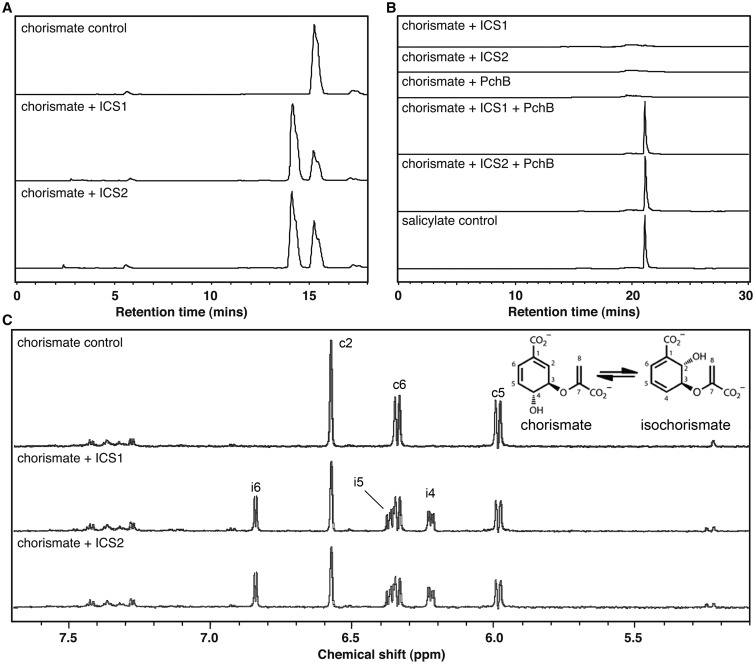
*Arabidopsis* ICS1 and ICS2 are both functional isochorismate synthases but not salicylate synthases. (**A**) HPLC analysis of product formation by ICS1 and ICS2 from chorismate. Chorismate is converted into isochorismate by both ICS1 and ICS2, when detected by absorbance at 274 nm. A reduction in substrate amount (chorismate peak at 15 min) is concomitant with an increase in product amount (isochorismate peak at 14 min). (**B**) Coupled HPLC assay for ICS activity demonstrates that neither ICS1 nor ICS2 possesses salicylate synthase activity. Incubation of the ICS assay mixture with a purified bacterial IPL (PchB) is necessary for salicylate formation, which confirms the identity of the product in (**A**), and restricts both ICS1 and ICS2 activity to isochorismate, rather than salicylate, formation. Analysis was performed using fluorescence detection with an excitation wavelength of 305 nm and an emission wavelength of 407 nm, and salicylate eluted after 21 min. (**C**) ^1^H NMR assay for ICS activity confirms that both ICS1 and ICS2 are functional ICSs, but lack salicylate synthase activity. Chorismate was incubated with ICS1 and ICS2 individually with product formation followed by ^1^H NMR. The spectrum was obtained after a 15 min incubation period at 20°C in 20 mM potassium phosphate buffer (pH 7.0) and 5 mM MgCl_2_. The abbreviations c and i correspond to signals for protons at the numbered positions on the chorismate and isochorismate molecules (see inset), respectively. Peaks were assigned as described in the ‘Experimental’ section.

Samples of recombinant hexahistidine-tagged variants of AtICS1 and AtICS2 were incubated with chorismate and the reaction products were analysed by HPLC. Samples of both His_6_-AtICS1 and His_6_-AtICS2 catalysed the conversion of chorismate into isochorismate, which was demonstrated by the appearance of a peak corresponding to isochorismate (HPLC retention time, 14 min) and a decrease in peak area for chorismate (retention time, 15 min; Figure 2A). No conversion occurred in reactions containing AtICS1 or AtICS2 protein samples that had previously been boiled for 3 min (results not shown). While ICS activity has been demonstrated previously for AtICS1 [10], this is, as far as we are aware, the first demonstration of *in vitro* enzyme activity of AtICS2.

Samples of purified His_6_-AtICS1 and His_6_-AtICS2 were tested for SAS activity. To do this, reactions were carried out in the presence or absence of an authentic IPL enzyme: PchB, a bacterial IPL from *P. aeruginosa*. Reaction products were analysed by HPLC but the eluate was monitored using a fluorescence detector, since this is a sensitive means of detecting SA. When PchB was included in the incubation together with either ICS1 or ICS2, an SA peak was detected (retention time, 21 min: Figure 2B). No SA was detected by HPLC analysis of reaction products of incubations that were not supplemented with PchB (Figure 2B). These results suggest that neither of the *Arabidopsis* ICS isozymes possess IPL activity.

To test further this conclusion, ^1^H NMR spectroscopy was employed in order to confirm the identity of the major product formed by the activities of AtICS1 and AtICS2 and to investigate if additional products are formed by the action of either *Arabidopsis* enzyme. NMR spectra were recorded after a 15 min incubation of chorismate with either AtICS1 or AtICS2, and compared with spectra of control incubations to which no enzyme was added (Figure 2C). With the addition of AtICS1 or AtICS2, the intensity of the chorismate signals was diminished, while isochorismate signals appeared. The reaction mix reached equilibrium after 15 min, with little change in the ratio of chorismate to isochorismate after this point, but no NMR signals corresponding to SA appeared. Taken together, these results show that neither of these *Arabidopsis* ICS proteins have IPL activity and, therefore, cannot be bifunctional SAS enzymes.

### AtICS1 and AtICS2 have similar catalytic properties

**Figure 3.**
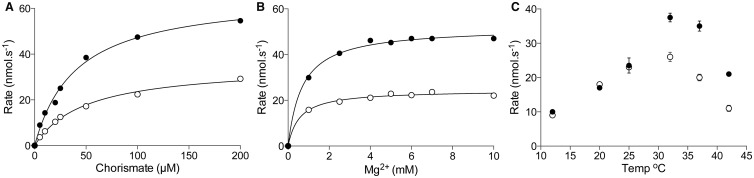
Kinetic characteristics of recombinant *Arabidopsis* ICS1 and ICS2. Effect on AtICS activity of (**A**) altering chorismate concentration, (**B**) Mg^2+^ concentration and (**C**) temperature. Assays were conducted using the coupled online fluorimetric method, using recombinant His_6_-ICS1/2 and His_6_-PchB. Closed circles, AtICS1; open circles, AtICS2.

To identify any differences in enzyme kinetics between AtICS1 and AtICS2, a coupled online assay (as opposed to a stopped assay, such as the HPLC and NMR assays previously used) for ICS activity was adopted. Purified His_6_-ICS1 or His_6_-ICS2 was incubated with chorismate and an excess of Mg^2+^ and PchB, and SA formation was measured by fluorescence detection. This enabled us to monitor the reaction kinetics in real time and to adjust more easily the assay conditions to test the response of the enzymes. Conversion of chorismate into isochorismate by both AtICS1 and AtICS2 obeyed Michaelis–Menten kinetics (Figure 3A), with *K*_m_ values for chorismate determined as 34.3 ± 3.7 µM (AtICS1) and 28.8 ± 6.9 µM (AtICS2) (Table 1). The catalytic efficiencies (*k*_cat_/*K*_m_) of AtICS1 and AtICS2 were, respectively, 1.11 ± 0.13  and 0.59 ± 0.15 µM^−1^ min^−1^, a difference which is primarily due to the higher turnover rate (*k*_cat_) for AtICS1 (Table 1). These results suggest that both AtICS1 and AtICS2 would be successful in competing for chorismate *in vivo*. The values for the *K*_m_ and *k*_cat_ for AtICS1 are consistent with those reported by Strawn et al. [10]. All previously studied bacterial and plant ICS enzymes require Mg^2+^ as a cofactor [10,37–39]. Both AtICS1 and AtICS2 required Mg^2+^, with a concentration of 4 mM required for maximal activity (Figure 3B). *K*_m_ values for [Mg^2+^] were 0.942 ± 0.069 mM for ICS1 and 0.55 ± 0.023 mM for AtICS2 (Table 1).

**Table 1:** Catalytic parameters for AtICS1 and AtICS2.

	AtICS1	AtICS2
*K*_m chorismate_ (µM)	34.3 ± 3.7	28.8 ± 6.9
*V*_max_ (nM s^−1^)	63.5 ± 2.4	28.3 ± 2.0
*k*_cat_ (min^−1^)	38.1 ± 1.5	17.0 ± 1.2
*k*_cat_/*K*_m_ (µM^−1^ min^−1^)	1.11 ± 0.013	0.59 ± 0.062
*K*_m Mg_ (mM)	0.942 ± 0.069	0.55 ± 0.023

The pH optima for activity of the two *Arabidopsis* isozymes differed, with the peak activity of ICS1 seen at pH ∼7.5 but that of ICS2 at pH 8 (Supplementary Figure S5). Strawn et al. [10] investigated the effect of temperature on AtICS1 activity and found a curious non-Gaussian response in which no appreciable increase in activity was found over the temperature range 0–30°C, with a drop in activity noted at higher temperatures. To investigate this further and to identify whether a similar phenomenon occurs when AtICS2 activity is assayed over a range of temperatures, the coupled online ICS assay was used and initial velocities were plotted as a function of temperature (Figure 3C). In our experimental system, ICS1 and ICS2 activity displayed a response to temperature that was more typical of most enzymes, with an approximate doubling in reaction rate per 10°C between 10 and ∼33°C, while at temperatures higher than this reaction rates declined (Table 1). In our experimental system, we did not see any evidence for a particularly strong activity of AtICS1, or AtICS2, at 4°C as suggested by Strawn et al. [10].

## Discussion

Here, we have extended our previous investigation of the relative expression of the two *ICS* genes in *Arabidopsis* and characterised the enzymatic activity of the purified recombinant proteins. We had previously found that *AtICS1* is not only induced by pathogen infection but also by SA itself, in a manner that is dependent on the classical defence regulator ‘Non-Expressor of Pathogenesis-Related Proteins 1’ [12,25]. In contrast, *AtICS2* expression is not enhanced by SA [25] or by pathogens [5,10]. In 2009, Yuan et al. [24] reported that seven different higher plants with sequence genomes possessed only a single *ICS* gene. We used sequence similarity searches of over 60 plant genomes currently available on Phytozome (phytozome.jgi.doe.gov) for genes encoding homologues of *Arabidopsis* ICS and again found that the norm is the presence of a single gene, the exceptions being in the Brassicales–Malvales, and soybean as previously identified. The existence of two *ICS* genes, most probably by gene duplication, in a plant such as *Arabidopsis* that has a relatively small genome is puzzling, but possession of two genes encoding ICS proteins with such highly similar sequences, structures and near-identical enzymological properties (as shown in the present study) suggests that this redundancy must confer some advantage on this plant. Duplication of *ICS* genes has also occurred independently in *Brassica napus* and soybean ([13,24] and data not shown). For poplar, which has only a single *ICS* gene, the primary *ICS* transcript undergoes alternative splicing to allow production of alternative isoforms of the enzyme [24]. This observation further underlines the possibility that there is some selective advantage, perhaps a conditional one, in having more than one form of ICS.

Our observations of expression of transgenes expressing *AtICS1* and *AtICS2* promoter–GUS fusion constructs indicated that whereas *AtICS1* expression, once induced, is widespread in the leaves, constitutive *AtICS2* expression is strongest in the veins and hydathodes. Hydathodes are secretory pores at the leaf margins that can be exploited as entry points into the vasculature by a range of microbial plant pathogens and food-contaminating human enteric pathogens (see ref. [40] and references therein). Interestingly, constitutive expression of several defence-related genes has been noted in the hydathodes and vasculature of *Arabidopsis*. For example, the SA-regulated *SUM3* gene, which encodes an SUMO modifier protein required for defence against plant pathogenic *Pseudomonas syringae*, is expressed in veins and hydathodes [41]. Expression of *myo-inositol 1-phosphate synthase 2*, which is required for basal resistance to bacterial, fungal and viral pathogens [42], was also observed predominantly in veins and hydathodes of *Arabidopsis* [43]. This suggests that although AtICS2 may not be critical for induced resistance to pathogens, it may play a role in maintenance of resistance to vascular invasion by microbes. It would, therefore, be interesting to see if *ics2* mutant *Arabidopsis* plants are more susceptible to infection by invasion by vascular pathogens

In *Arabidopsis*, there are differences between the regulatory DNA sequences of the *AtICS1* and *AtICS2* genes; specifically potential methylation sites are present in the promoter region of *AtICS2* but are absent from the corresponding region of the *AtICS1* gene (Supplementary Figure S1). Such differences in methylation, which is known to affect transcription [44], may explain the contrasting expression pattern of AtICS2, which appears to be constitutively expressed, and AtICS1, which is inducible. However, the possibility cannot be dismissed that *AtICS2* may only appear to be constitutively expressed. To date, the expression of *AtICS2* has been investigated only in response to exogenous SA treatment and pathogen attack. It is conceivable that if a wider range of stimuli were applied, or if its expression were examined over the entire course of plant development, *AtICS2* transcription might be found to vary under some specific set of conditions. Whether or not *AtICS2* is always expressed constitutively, its expression was increased in the *gun4* mutant [31]. This deregulation in *gun4* plants suggests that *AtICS2* gene expression is under negative regulatory control by the network governing the co-ordination of nuclear and plastid gene expression, which may relate to the plastidic location of both ICS isozymes in *Arabidopsis*.

We have shown with three different assay systems that AtICS1 and AtICS2 are both able to catalyse the interconversion of chorismate and isochorismic acid. To our knowledge, this was the first direct demonstration of the ICS activity of AtICS2. The ICS activity of ICS2 is slightly lower than that of ICS1, but otherwise the catalytic characteristics of the two enzymes are very similar, as are their structural characteristics, which resemble those of the *E. coli* ICS, EntC [17]. The kinetic properties of AtICS1 and AtICS2 do not appear to show any characteristics that are strikingly different in nature from previously investigated bacterial ICS enzymes. For example, in contrast with the observations of Strawn et al. [10], who found no significant difference in the activity of AtICS1 between 4°C and ∼37°C, we found that activity increased with temperature.

It had been shown previously in transgenic *Arabidopsis* plants that constitutive expression of a recombinant bifunctional SAS created by translational fusion of the *P. aeruginosa* pchA and pchB coding sequences resulted in constitutive SA accumulation, regardless of whether the SAS was expressed in the chloroplast or cytosol [18]. It has also been shown that expression of separate ICS and IPL enzymes in the chloroplasts of transgenic tobacco plants engendered constitutive SA accumulation [19]. Taken together, these studies showed that either biosynthetic ‘approach’ to SA biosynthesis could, in principle, work *in planta*. However, importantly we have shown here that neither AtICS1 nor AtICS2 possesses detectable IPL activity. Thus, SA biosynthesis in *Arabidopsis* follows a pathway analogous to that in *P. aeruginosa*, which possesses distinct ICS and IPL enzymes [16] rather than that of *Y. enterocolitica*, which has a bifunctional SAS [14,15]. It may be worth noting that SAS-expressing transgenic *Arabidopsis* plants were reported to possess a stunted phenotype [18]. While this might have resulted simply from the production of phytotoxic levels of SA, this seems unlikely since the increases in SA levels in the transformed lines were relatively modest, and instead it was proposed that expression of an SAS somehow disrupts the regulation of the chorismate pool, with deleterious consequences for other chorismate-dependent biosynthetic pathways located in the chloroplast, such as for aromatic amino acids or folate. In contrast, Verberne et al. [19], using the approach of constitutively expressing separate ICS and IPL enzymes in the chloroplast, created plants with markedly increased levels of SA but with no reported change in phenotype. This appears to support the hypothesis of Mauch et al. [18] that an SAS may disrupt plastid-based biosynthetic pathways, and it may perhaps explain why *Arabidopsis* has not evolved to possess an SAS. In the meantime, although *Arabidopsis* must possess one or more proteins possessing IPL activity, the identity of this enzyme or enzymes remains unknown.

## Abbreviations

*At*: Arabidopsis thaliana
*gun4*: *genomes uncoupled 4*
GUS: β-*glucuronidase*
HPLC: high-performance liquid chromatography
HR: hypersensitive response
ICS: isochorismate synthase
IPL: isochorismate pyruvate lyase
IPTG: isopropyl β-d-thiogalactopyranoside
NMR: nuclear magnetic resonance
PBD: Protein Data Bank
PDA: photodiode array
SA: salicylic acid
SAS: SA synthase
*sid2*: *salicylic acid induction-deficient 2*
TEV: tobacco etch virus
TFA: trifluoroacetic acid.
